# Burden of COVID-19 on mental health of older adults in a fragile healthcare system: the case of Nigeria: dealing with inequalities and inadequacies

**DOI:** 10.1017/S1041610220001726

**Published:** 2020-08-12

**Authors:** O. Baiyewu, O. Elugbadebo, Y. Oshodi

**Affiliations:** 1 Department of Psychiatry, College of Medicine, University of Ibadan, Ibadan, Nigeria; 2 Department of Psychiatry, College of Medicine, University of Lagos, Lagos, Nigeria

When the World Health Organization declared the COVID-19 a pandemic, concerns were expressed on the possible effect of the virus on countries in Africa with fragile health systems and poor indices of human development (Makoni, 2020). For example, prior to the pandemic, per capita health expenditure and the ratio of healthcare personnel to the general population were low (Institute of Health Matrix and Evaluation, 2020); moreover, there is a poor political will to improve health care generally. To compound this in Nigeria is the fact that insurgence is common, especially in the Northeastern part of the country, and that has produced many internally displaced persons who live in various camps. In addition, there are many street children begging for alms partly due to religious and cultural reasons; these again are more common in Northern Nigeria. It, therefore, looks like the ground is fertile for a rapid spread of COVID-19.

Be that as it may, it appears that the rate of infection and mortality is relatively low in Nigeria; however, it should be noted that only suspected persons and their contacts are tested for economic logistic reasons. As of June 9, 2020, Nigeria had tested 79,948 persons out of which 12,801 persons were confirmed with COVID-19 and 361 deaths (Nigeria Center for Disease Control, 2020); thus, death rate was 0.45%; from the sample was 0.45% and 2.8% from confirmed cases, compared with 5% death rate in confirmed cases globally. It could be argued that Nigeria is not screening enough and some true cases could have been missed. Death is higher in people above 50 years and those with existing comorbidities (Nigeria Center for Disease Control, 2020). The reason why mortality is relatively lower in Nigeria and other countries in Sub-Saharan is not clear but one could speculate that because median age in Nigeria is 18 years while in Europe, it is 46 and 47 years for Germany and Italy, respectively (worldometer, n.d.); death rate is lesser because of the relatively younger population.

Health care in Nigeria is concurrently the responsibility of the federal government and 36 subnational state governments. Health care is delivered by university teaching hospitals and other specialist hospitals at the tertiary level, general hospitals at the secondary level, and primary health center at the local level all run by the government. Many private hospitals with varying degrees of skills and competencies operate at levels approximate to these three levels. Only the federal government and few states like Lagos had the adequate infrastructure to respond effectively to the challenges of COVID-19 virus when it started. In 2014, the Ebola virus spread to Nigeria from a neighboring country Liberia, through air transportation. Major cases were in Lagos and national spread was largely prevented through proactive measures taken by health authorities in Lagos state. However, COVID-19 has not been that easy to handle and it has spread to 35 of the 36 states but about 46% of cases are in Lagos. Lagos has the largest economy among the Nigerian subnational states; poverty headcount was lowest in Lagos at 4.5% compared with the national average of 40.1% (National Bureau of Statistics, 2019). Consequently, there are differences in the provision of health facilities by different states and Lagos appears to take the lead. Another important point is that the National Health Insurance of Nigeria covers only about 4% of the population (Okebukola and Brigger, 2016) most of the enrollees are employees of the government, and there is very poor coverage for retirees and older adults. Most Nigerians, especially the older adults, have to depend on out of pocket payment for health services as reported recently in Northern Nigeria (Azubuike *et al*., 2020). This method of healthcare provision leads to impoverishment when such people are faced with chronic illnesses like cancer (Gottret and George, 2017). Even more germane to this discourse is the fact that the pathway to health care in Nigeria (more so for the rural elderly and mental health in general) is via the alternate medicine and spiritual homes route (Gureje *et al*., 1995).

With such a parlous situation on the health care of older adults in Nigeria, the warning of the WHO that the effect of COVID-19 on the countries in Africa would be grim is understandable. (Makoni, 2020).

The effects of COVID-19 on mental health of the older adults in Nigeria can be grouped into a number of subheadings. Many psychological effects follow the directives of government on personal hygiene, safe distancing, and lockdowns.

## The fear of contagion

Based on information in the media originating from health institutions that older adults with comorbid conditions are liable to die faster from COVID-19, caregivers prevent them from receiving visitors. Family members not resident with older adults reduce the frequency of their visits for the same reason, even when they wish to, there are restrictions on this due to lockdown by the government as intercity or interstate travels are discouraged except for medical workers and food suppliers. A case in point was a man whose mother has stroke and lives some 65 km away, son was worried that he could inadvertently carry virus to mother and when mother demanded he should visit, the lockdown made that impossible.

## Access to health care

The COVID-19 pandemic has changed the face of health care in Nigeria. The fear of contagion and the need to protect other patients and health workers from infection have led hospitals and clinics to limit services to emergency cases mainly, and many older adults with chronic conditions like arthritis, cancers, diabetes mellitus, stroke, and dementia are unable to attend regular clinics but are seen only when emergency situations arise. Nonemergency surgical procedures even if disabling are put on hold in many government hospitals; however, private hospitals provide various levels of care though limited because of lockdown and need to protect other patients and staff. In a few cases, older adults or their caregivers can call their healthcare providers, but such arrangements are informal and few. The worst hits are the rural areas where ab initio healthcare provisions are precarious.

## Older adults who test positive for COVID-19

People who test positive for COVID-19 are required to go into isolation and this is a major concern for the older adults. Brooks *et al*. (2020) reviewed reaction to quarantine for various epidemics in general population cohorts and reported that quarantine experiences include stigma, frustrations and boredom, fear of infection, and worries about finances and inadequate supplies while in quarantine. Observations by one of us (YO) is that those over the age of 60 years who need to go into isolation exhibit these symptoms and, in addition, they are often anxious and apprehensive about what the future holds, when they are informed they are COVID-19 positive. The degree of anxiety is worse in the more educated group who are diagnosed with comorbid illnesses; as information is readily available that such people have a higher risk of mortality. YO leads the Lagos psychological response team for COVID-19 treatment center as of 30 April, there were over 200 COVID-19 positive persons who had gone through the psychological support service while in isolation and about 7% of them are 60 years or older. Roger *et al.* (2020) reviewed studies on coronavirus infection which included severe acute respiratory syndrome and Middle East respiratory syndrome compared with COVID-19. Data on COVID-19 were sparse but extrapolating the similarities in symptoms suggests that delirium, confusion, and agitation would be common in the intensive care units while depression, anxiety, and post-traumatic stress disorder would be common at the post-recovery phase. The post-recovery phase of COVID-19 should be of interest to researchers.

## Care of people living with dementia

These persons are subjected to unique difficulties during this pandemic period. There is an ongoing study on the effect of COVID-19 pandemic on caregivers of persons with dementia by our group. Initial impression from the study indicates that caregivers prevent visitation to people with dementia. Individuals with dementia find it difficult to comprehend what COVID-19 is all about. They ask friends, carers, and family members who do not visit regularly. Due to cognitive impairment, some repeat questions even when the situation had been adequately explained; some complain that they do not attend clinics as regularly as they did in the past.

Caregivers are worried about getting medicines for their wards since hospitals limit treatment of non-emergencies, even though pharmacies are open, there might be no prescriptions. The next is how to handle behavioral and psychological symptoms of dementia, delirium, and other comorbid conditions when there is little or no access to healthcare providers. However, in a few centers, family members could call in to seek assistance.

Most older adults with psychiatric morbidities are treated by nonmental healthcare providers in various hospitals and health centers across the nation. Psychogeriatric services are available in seven centers across the nation and these are manned by multidisciplinary treatment groups, often led by psychiatrists who are members of the Geriatric Psychiatric Section of the Association of Psychiatrists in Nigeria. The section is an affiliate of the International Psychogeriatric Association. In a survey of five of these clinics, dementia was the most common diagnosis (43%) followed by schizophrenia and related disorders (24%) and depression (15%) (Baiyewu *et al*., 2015). These clinics are run specifically for older adults requiring mental health services. Incidentally, none of these centers has an admission ward specifically designated for older adults and when older adults with mental health conditions need admission, they are admitted into general adult psychiatric facilities.

## Long-term care

Nursing homes are few and unpopular in Nigeria, with little or no support from the government. Only a few are directly run by the government, while others are run by NGOs and religious organizations. Most of these are meant for destitute and are often run as charities. There are a few private nursing homes now for people who pay out of pocket, but regulations on these establishments are weak. In a small study in two of these charity-based homes, a couple of years back, it was reported that 48% of residents had dementia and 17% depression (Baiyewu *et al*., 1997); there are no recent studies and the situation might have changed now and no information on the effect of COVID-19 on older adults in these facilities is available. Even though we have no information on death rate in nursing homes, various anecdotal reports from countries in Europe indicate increased death rate in the very old and those with dementia who are likely to be residents of nursing homes (Dichter *et al*., 2020; Wang *et al*., 2020). However, in a retrospective study, Bianchetti *et al.* reported that patients who died in a COVID-19 treatment facility in Northern Italy are likely to be older and have dementia. More also, the severity of dementia as measured by the Clinical Dementia Rating scale (Hughes *et al*. 1982) is related to a higher odds ratio for mortality. In a comparative mortality study between two centers in Ibadan, Nigeria and Indianapolis, USA, more significantly more older person free of dementia died in Nigeria while more with dementia still died though not significantly different when rates in the two sites were compared (Perkins *et al*., 2002).

## Intervention processes

It has been always gloom and doom with respect to COVID-19 infection in Nigeria. Nationally, there has been a massive upgrade of health facilities principally geared towards the prevention of spread of the virus. Almost on a daily basis, there was a briefing on the status of the infection by the Presidential Task force which includes the Center for Disease Control, the Ministry of Health, and many other ministries and agencies. Regular information is given on the number of new infections per state and what needs to be done to flatten the curve. A website by the Nigeria Center for Disease Control gives regular information at the beginning of COVID-19 infection, only two centers had reliable diagnostic facilities, but that has rapidly gone up to 22 as of June 2020 and in addition, many isolation centers have been established through various government agencies business community and NGOs. Palliatives were also given to the poor in society by the government and NGOs.

Aside from these, some centers developed their own programs albeit of limited value, one of such by the University College Hospital in Ibadan encourages geriatric patients to remain at home while family can call dedicated telephone lines to ask for support as regards medication use as well as for other health-related matters. That was the program for 95% of patients on their database, but the rest 5% who were thought to be vulnerable were seen in the hospital with doctors clad in PPE (Adebusuyi *et al*., 2020). Psychogeriatric patients in the same hospital enjoyed similar support and families received support particularly for patients with behavioral and psychological symptoms of dementia through telephone counseling and were seen by special arrangements. However, this type of arrangement is limited to a facility and there was no national coordination, though other centers probably had their own programs.

## Thoughts about post-pandemic era

Up till the onset of the pandemic, there was a little political will to provide adequate and accessible health care to the Nigerian citizenry. The political and business class prefers to go abroad to attend to their health needs except in unavoidable emergency situations. It would appear that COVID-19 has forced a new normal on us. The virus is no respecter of class or political inclination and a few infected members of the political class who ordinarily would have traveled abroad for medical care found that impossible and had to seek treatment at home. This led to the rapid expansion of treatment and isolation centers that were equipped to handle the pandemic. However, there is a suspicion that the lessons learned might lead only to the establishment of high-class medical centers like the type they visit abroad without addressing the healthcare needs of the citizenry. Paul *et al*. (2020) discussed the nexus between COVID-19, local, national, and global health. The authors advocated a shift from the “Pasteurian paradigm” to a holistic model of health care. The Pasteurian model envisions that each disease is due to one pathogen; thus, the cure for each disease is by targeting the responsible pathogen. However, the traditional frontiers between communicable and noncommunicable diseases are now blurred by evidence of “biosocial contagion” (Seeberg and Meinert 2015). This is akin to the organic/nonorganic dichotomy in mental health which has now been jettisoned in the DSM-5 diagnostic model. In a nutshell treatment of COVID-19 should be biopsychosocial, more so for the older adults who have higher chances of comorbid illnesses.

## Conclusion and recommendation

1. It is necessary to increase healthcare expenditure by all agencies of the government as well as by the business community. Nigeria spent $71 per capita on health in 2016, the estimated 2017 GDP per capita was $5252 (Institute of Health Matrix and Evaluation, 2020) this is obviously low. The United Nations Department of Economic and Social Affairs reported that most older adults in Sub-Saharan Africa depend on family sources to fund consumption for health and social care; while in the Euro-America regions, it is public transfers (pension and social care programs) that the older adults depend on (World Population Ageing, 2019).

Other ways of funding should be tapped into, for example, PricewaterhouseCooper (2019) reported that Nigerians in diaspora remitted 23.63 billion dollars in 2018, mostly to support the family for education, feeding, and health. No information is available on what amount out of this money got to the older adults and if there is variation in regional distribution. However, it is important to look into how part of such funds could be used to uplift health care for older adults through the provision of health infrastructure and private health insurance policies targeted at the special needs of the older adults in the Nigerian context.

1. There is a need for improvement in care programs for the elderly including mental health and social support components. This may include home care support options and strategies for home delivery of groceries and medications, particularly for those who have no families.
2. Long-term care needs attention in infrastructure, service provision, and appropriate regulation.
3. In centers in high-income countries, innovative methods in health delivery like telemedicine and telepsychiatry are commonplace (Wosik *et al*., 2020). While there could be some limitations in applying some types of technology in Sub-Saharan Africa, a good part of it is implementable if properly planned. The most common method of virtual intervention in the mental health of older adults in Nigeria is by telephony; however, during COVID-19 lockdown, it was possible to hold conferences and journal clubs via Zoom in a number of centers, such can easily be upgraded to involve consultation between specialists and primary health workers.
